# The Size and Shape Effects on the Melting Point of Nanoparticles Based on the Lennard-Jones Potential Function

**DOI:** 10.3390/nano11112916

**Published:** 2021-10-30

**Authors:** Anwar Al Rsheed, Saad Aldawood, Omar M. Aldossary

**Affiliations:** Department of Physics and Astronomy, College of Science, King Saud University, P.O. Box 2455, Riyadh 11451, Saudi Arabia; anwaromr4@gmail.com (A.A.R.); SDawood@ksu.edu.sa (S.A.)

**Keywords:** lennard-jones potential function, cohesive energy, melting point, nanoparticles, shape factor

## Abstract

A model is proposed to calculate the melting points of nanoparticles based on the Lennard-Jones (L-J) potential function. The effects of the size, the shape, and the atomic volume and surface packing of the nanoparticles are considered in the model. The model, based on the L-J potential function for spherical nanoparticles, agrees with the experimental values of gold (Au) and lead (Pb) nanoparticles. The model, based on the L-J potential function, is consistent with Qi and Wang’s model that predicts the Gibbs-Thompson relation. Moreover, the model based on the non-integer L-J potential function can be used to predict the melting points $T_{m}$ of nanoparticles.

## 1. Introduction

Melting point is a thermal property that depends on the size of materials and was first observed in 1954 [1]. Many experimental methods have been used to measure the melting point of nanoparticles, such as transmission electron microscopy (TEM) [2], differential scanning calorimetry (DSC) [3], nanometer scale calorimetry [4], differential thermal analysis (DTA) coupled to thermal gravimetric analysis (TGA) techniques [5], etc. It has been found that the melting points of metals with different shapes [1,2,3,4,5,6,7,8,9,10] and semiconductors [11] decrease as their thickness decreases. The first theoretical description of the size-dependent melting point of nanoparticles was in 1909 by the relation known as Gibbs-Thompson relation [12], which takes the following form:

(1) $$T_{m}=T_{mbulk}\left(1-\frac{C}{D}\right)$$

where $T_{m}$ and $T_{mbulk}$ are the melting points of the nanoparticle and bulk material, respectively, C is a constant that depends on the material of the nanoparticle, and D is the nanoparticle thickness (e.g., the diameter of spherical nanoparticles). The Gibbs-Thompson relation shows that the melting points of nanoparticles are linearly proportional to the reciprocal of the nanoparticle thickness. Many experimental works [11,13,14,15,16] have showed that the melting points of nanoparticles follow the Gibbs-Thompson relation.

There are three different theoretical thermodynamic models that describe the constant C in the Gibbs-Thompson relation: The homogeneous liquid-drop model (LDM) considers both the solid and liquid states of nanoparticles in a homogeneous phase [5,12,17]; the liquid shell nucleation model (LSN) considers the equilibrium of the solid core and thin liquid shell in nanoparticles [17,18,19]; and the liquid nucleation and growth model (LNG) supposes the surface of nanoparticles melts first and then grows into the entire solid [17,20,21].

Many models were proposed to calculate the melting point of non-spherical shape nanoparticles that follow the Gibbs-Thompson relation. A new parameter was introduced to consider the non-spherical shape called the shape factor which is defined as the ratio of the surface area of non-spherical nanoparticles to the surface area of spherical nanoparticles that have similar volume [22,23]. The shape factor was introduced in many models, such as Qi and Wang’s model that based on the surface-to-volume ratio [24,25], the surface-area-difference (SAD) model where the cohesive energy of a nanoparticle that consists of n atoms is equal to the increased surface energy due to the surface area difference of n atoms and the nanoparticle [26], and a nonlinear lattice type sensitive model which considers the lattice structure and surface-to-volume ratio of the nanoparticle [27,28]. Other models found that C in Equation (1) is a size-dependent parameter, such as Zhang et al.’s model where surface atoms and interior atoms have different effects upon the melting point [29] and Chen et al.’s model that is based on Gibbs free energy [30].

It is found that the melting point of nanoparticles is linearly proportional to their cohesive energy [31], where the cohesive energy of nanoparticles is equal to the sum of bond energies of  n atoms in an equilibrium configuration [32,33]. The bond energy between two atoms in the nanoparticles can be calculated using many different potential functions such as the Lennard-Jones (L-J) (12-6) potential function [34,35], non-integer L-J potential function [32], Mie-type Potential function [36], Morse potential function [37], and generalized Morse potential function [33]. The cohesive energy and melting point of nanoparticles based on the upper potential functions does not consider the shape of nanoparticles.

The L-J (12-6) potential function is a well-known semi-empirical formula that has two physically defined terms: The first term represents the empirical Pauli repulsive potential, whereas the second term represents the dipole-dipole attractive potential [38]. However, the Morse potential was obtained by Taylor expansion of an unknown from of the potential function around an equilibrium distance between two neighbor atoms [39]. Moreover, the L-J (12-6) potential function does not have adjustable parameters, unlike the Morse potential function and the other potential functions. Additionally, the pair interaction potentials (e.g., L-J (12-6) potential) are accepted to describe the behavior of metals [40]. An extended L-J embedded-atom potential model was used to describe the physical properties of FCC metals [41] and Thorium-doped Tungsten Crystals [42]. The LAMMPS molecular dynamics simulation method was used to calculate the cohesive energy of some metals based on the L-J (12-6) potential where the discrepancy between the calculated and experimental values was less than 0.1% [43]. Moreover, the same method based on the L-J (12-6) potential was used to calculate the bulk melting point of Fe, Ni, Pb, and Cr with very small discrepancy if the parameters of the L-J (12-6) potential were normalized [44]. Therefore, the aim of the present work is to build a model using the L-J (12-6) potential function to calculate the melting point of metallic nanoparticles considering the shape and structure of nanoparticles, and the effect of the dangling bonds of the surface atoms of nanoparticles.

The paper is organized as follows. In Section 2, we describe the theory and model to calculate the melting point of a nanoparticle based on L-J (12-6) potential function considering the shape and structure of nanoparticles and dangling bonds of the surface atoms. Section 3 is about the results of the melting point based on the present model and compared to other models such as Qi and Wang’s model [24] and Qi et al.’s model [34], and with the available experimental data. Finally, in last section, discussions about the validity of the present model are presented.

## 2. Theory and Model

The total cohesive energy of nanoparticles is the sum of the total interior and the total superficial parts [45]:

(2) $$nE_{a}=\left(n-N\right)E_{I}+\frac{1}{2}NE_{S}$$

where n and N denote the total number and surface number of atoms, respectively, E is the cohesive energy per atom, and the subscripts *a*, *I*, *S*, mean whole, interior, and surface of the nanoparticle, respectively. The factor 1/2 in the second term of the right side of Equation (2) is due to the bonds of the surface atoms of the nanoparticles being dangling bonds [25,46].

Dividing Equation (2) by the total number of atoms n and using the definition of the surface-to-volume atomic ratio $s_{n}=N/n$ [47], the following is obtained:

(3) $$E_{a}=\left(1-s_{n}\right)E_{I}+\frac{1}{2}s_{n}E_{S}$$

In the present model, the nanoparticles are considered in a spherical shape with a diameter equal to D. Thus, the total number of atoms in the nanoparticle equals the ratio of the nanoparticle volume to the atomic volume multiplied by the volume packing factor $P_{L}$ as follows:

(4) $$n=P_{L}{\left(\frac{D}{d}\right)}^{3}$$

where d is the atomic diameter.

The surface number of atoms in the nanoparticle equal the ratio of the surface area of the nanoparticle to the cross-section area of the atom multiplied by the surface packing factor $P_{f}$ as follows:

(5) $$N=4P_{f}{\left(\frac{D}{d}\right)}^{2}$$

Equations (4) and (5) can be used to rewrite the surface-to-volume atomic ratio $s_{n}$ as a function of the total number of atoms n in the nanoparticle as follows:

(6) $$s_{n}=\frac{4P_{f}}{P_{L}^{\frac{2}{3}}n^{\frac{1}{3}}}$$

The surface-to-volume atomic ratio parameter $s_{n}$ as shown in Equation (6) is a size-dependent parameter because it is $\propto n^{-\frac{1}{3}}$. The parameter $s_{n}$ also depends on the structure of the nanoparticle because it depends on the volume and surface packing factors. The volume and the surface packing factors of different cubic structures (SC, BCC, and FCC) are calculated as described in Safaei et al.’s paper [27] and summarized in Table 1. The bulk limit (n→∞) of the parameter $s_{n}$ is $s_{\infty }=\underset{n\to \infty }{\lim}s_{n}\to 0$, which means no shape effect for bulk materials.

The cohesive energy of the interior nanoparticle $\left(n-N\right)E_{I}$ and the surface nanoparticle $NE_{S}$ are equal to the sum of the bond energies of the interior atoms and the bond energies of the surface atoms in the nanoparticle, respectively. As result, the cohesive energy of the nanoparticle per atom can be calculated using the following formula:

(7) $$E_{a}=\frac{1}{2}\left\{\frac{1-s_{n}}{n-N}\overset{n-N}{\underset{i=1}{\sum }}\overset{n}{\underset{\begin{array}{c} j=1\\ j\neq i \end{array}}{\sum }}U_{ij}+\frac{s_{n}}{2N}\overset{N}{\underset{i=1}{\sum }}\overset{n}{\underset{\begin{array}{c} j=1\\ j\neq i \end{array}}{\sum }}U_{ij}\right\}$$

where $U_{ij}$ is the bond energy between two atoms in the nanoparticle. The bond energy $U_{ij}$ can be calculated using the L-J (12-6) potential function [38,48]:

(8) $$U_{ij}=D_{0}\left\{{\left(\frac{r_{0}}{r_{ij}}\right)}^{12}-2{\left(\frac{r_{0}}{r_{ij}}\right)}^{6}\right\}$$

where $D_{0}$ represents to the depth of the potential, $r_{ij}$ and $r_{0}$ are the relative distance between the *i*th and *j*th atoms and distance between the nearest atoms in the nanoparticle, respectively. The first term on the L-J (12-6) potential function represents to the short-range Pauli’s repulsive potential, where the second term represents to the long-range dipole attractive potential [38,48].

The cohesive energy per atom of the nanoparticle based on the L-J (12-6) potential function is written as:

(9) $$E_{a}=\frac{D_{0}}{2}\left\{\frac{A_{12}}{r^{*12}}-2\frac{A_{6}}{r^{*6}}\right\}$$

where $r^{*}=r/r_{0}$ is the reduced nearest distance between two atoms,

(10a) $$A_{6}=\left(1-s_{n}\right)A_{6}^{I}+\frac{1}{2}s_{n}A_{6}^{S}$$

and

(10b) $$A_{12}=\left(1-s_{n}\right)A_{12}^{I}+\frac{1}{2}s_{n}A_{12}^{S}$$

are the potential parameters, where

(11) $$A_{k}^{I}=\frac{1}{n-N}\overset{n-N}{\underset{i=1}{\sum }}\overset{n}{\underset{\begin{array}{c} j=1\\ j\neq i \end{array}}{\sum }}{\left(\frac{1}{a_{ij}}\right)}^{k}$$

(12) $$A_{k}^{S}=\frac{1}{N}\overset{N}{\underset{i=1}{\sum }}\overset{n}{\underset{\begin{array}{c} j=1\\ j\neq i \end{array}}{\sum }}{\left(\frac{1}{a_{ij}}\right)}^{k}$$

where k=6 and 12, and $a_{ij}=\left(r_{ij}/r\right)$. The parameters $A_{k}^{I}$ and $A_{k}^{S}$ represent to the contribution of the interior atoms and surface atoms in the value of the potential parameters, respectively.

**Figure 1:**
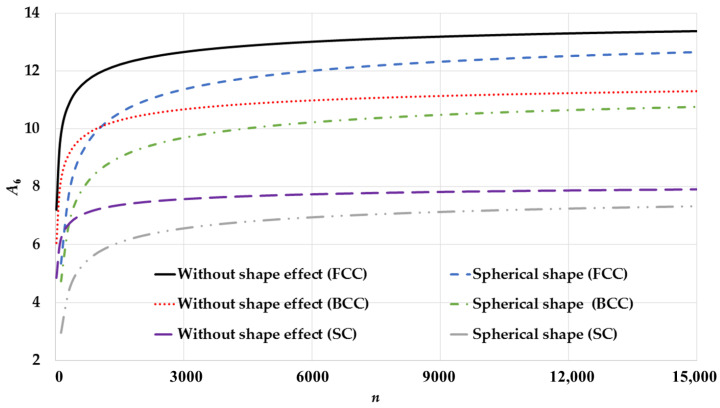
Size-dependent of the potential parameter $A_{6}$ as function of n of spherical nanoparticles in different cubic structures: SC, BCC, and FCC and compared to without shape effect case that is based on Qi et al. model [34].

**Figure 2:**
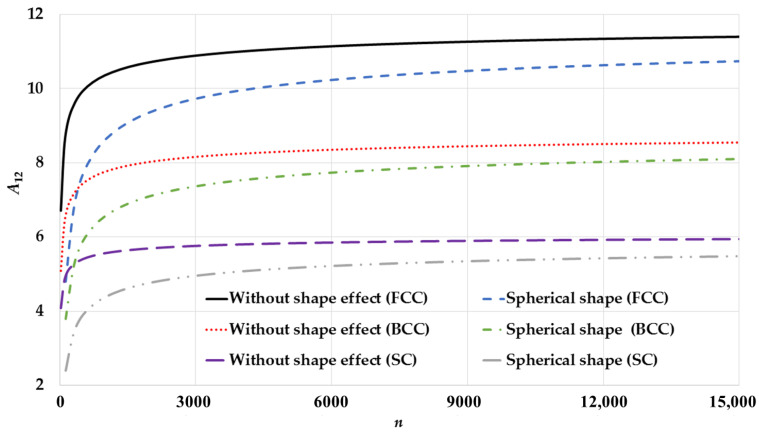
Size-dependent of the potential parameter $A_{12}$ as function of n of spherical nanoparticles in different cubic structures: SC, BCC, and FCC and compared to without shape effect case that is based on Qi et al. model [34].

The potential parameters are calculated based on the present model using Equations (10a) and (10b) for spherical nanoparticles and based on Qi et al.’s model [34] that does not consider the shape effect for different cubic structures (SC, BCC, and FCC) as seen in Figure 1 (for $A_{6}$) and Figure 2 (for $A_{12}$). As shown in the figures, the calculated potential parameters $A_{6}$ and $A_{12}$ for spherical nanoparticles are size-dependent. The calculations show that the growth of the potential parameters as a function of number of atoms n for spherical nanoparticles based on the present model is slower than the Qi et al.’s model [34]. The surface-to-volume atomic ratio parameter $s_{n}\propto n^{-\frac{1}{3}}$ in the present model, as shown in Equations (10a) and (10b), is behind the slow growth of the potential parameters. When the nanoparticle has a very small number of atoms then the contribution of the surface atoms is larger than the interior atoms in the calculation of the potential parameters. Therefore, the values of the potential parameters of spherical nanoparticles are smaller than those which do not consider the shape effect. As the number of atoms in nanoparticles increases, the value of the parameter $s_{n}$ decreases by factor $\propto n^{-\frac{1}{3}}$, so the contribution of the interior atoms increases by factor equals to $\left(1-s_{n}\right)$, and the contribution of the surface atoms decreases by factor equals to $s_{n}$ in the calculation of the potential parameters. Therefore, the growth of the potential parameters as a function of the number of atoms n for spherical nanoparticles is slow.

$A_{k}^{’}$ is the corresponding bulk value of $A_{k}$ (when n→∞ or N/n→0), which it can be obtained as follows:

(13) $$A_{k}^{’}=\underset{n\to \infty }{\lim}\left\{\left(1-s_{n}\right)A_{k}^{I}+\frac{1}{2}s_{n}A_{k}^{S}\right\}=\underset{n\to \infty }{\lim}\frac{1}{n}\overset{n}{\underset{i=1}{\sum }}\overset{n}{\underset{\begin{array}{c} j=1\\ j\neq i \end{array}}{\sum }}{\left(\frac{1}{a_{ij}}\right)}^{k}$$

The bulk value of $A_{k}$ (k=6 and 12) for different cubic structures is summarized in Table 2.

The equilibrium value of $r^{*}$ can be obtained by minimizing Equation (9) to be as: $r_{0}^{*}={\left(A_{12}/A_{6}\right)}^{1/6}$. Therefore, the cohesive energy per atom in the equilibrium configuration is [38]:

(14) $$E_{a}=-\frac{D_{0}}{2}\frac{{\left(A_{6}\right)}^{2}}{A_{12}}$$

The relative cohesive energy of the nanoparticle is defined as the ratio of the cohesive energy per atom of the nanoparticle to the corresponding cohesive energy of the bulk material $E_{0}$:

(15) $$\frac{E_{a}}{E_{0}}=\frac{P_{0}}{2}\frac{{\left(A_{6}\right)}^{2}}{A_{12}}$$

where $P_{0}=2A_{12}^{’}/{\left(A_{6}^{’}\right)}^{2}$.

The melting point $T_{m}$ of nanoparticles is linearly proportional to cohesive energy as follows [49]:

(16) $$T_{m}=0.032\frac{E}{k_{\beta }}$$

where $k_{\beta }$ is the Boltzmann constant, then the ratio of melting point of nanoparticle $T_{m}$ with *n* atoms to the bulk melting $T_{mbulk}$ equals to the relative cohesive energy of the nanoparticle:

(17) $$\frac{T_{m}}{T_{mbulk}}=\frac{P_{0}}{2}\frac{{\left(A_{6}\right)}^{2}}{A_{12}}$$

## 3. Results and Discussion

**Figure 3:**
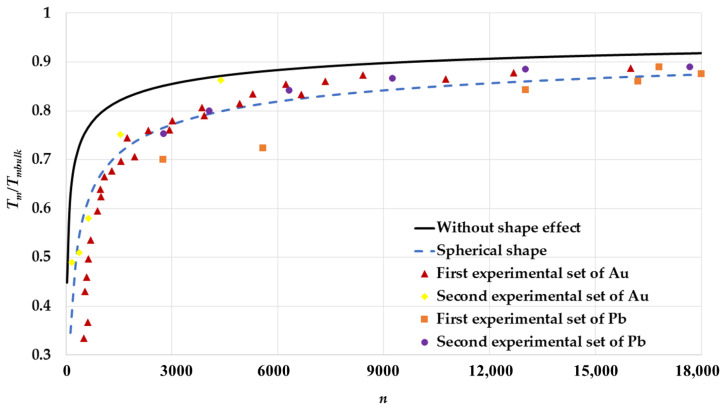
The variation of ratio $T_{m}/T_{mbulk}$ as function of the number of atoms n of spherical nanoparticles in an FCC structure is compared to without shape effect case that is based on Qi et al. model [34], two different experimental sets of Au nanoparticles: The first experimental set of Au [2] and the second experimental set of Au [5], and two different experimental sets of Pb nanoparticles: The first experimental set of Pb [6] and the second experimental set of Pb [7].

The melting points of spherical nanoparticles in an FCC structure are calculated based on the L-J potential function using Equation (17) and compared to Qi et al.’s model (without shape effect) [34] as seen in Figure 3. It is found that the calculated melting points of nanoparticles that do not consider the shape effect (Qi et al.’s model) are higher than spherical nanoparticles (present model). Since Qi et al.’s model does not consider the shape effect, it treats the surface atomic bonds and the interior atomic bonds in the same manner. However, the present model considers that if the shape of the nanoparticles is spherical, then the parameter $s_{n}\propto n^{-\frac{1}{3}}$. As the size of a spherical nanoparticle reduces, then the parameter $s_{n}$ becomes large, so the contribution of the surface atomic bonds is larger than the interior atomic bonds in the total energy of the nanoparticle, which makes its core less stable. This result agrees with the idea that the melting process begins on the surface of nanoparticles [29]. Therefore, the calculated melting point that considers the shape of a nanoparticle as a spherical shape is smaller than that which does not consider the shape effect. Thus, the melting points of spherical nanoparticles decrease faster as their size decreases.

The calculated melting points of nanoparticles in an FCC structure are also compared to two sets of experimental data of melting points of Au nanoparticles [2,5] (the mass density is $\rho =18.4\;g/{\mathrm{cm}}^{3}$ and $T_{mbulk}=1337.3\;K$ [50]) and two sets of experimental data of melting points of Pb nanoparticles [6,7] (the mass density is $\rho =11.3\;g/{\mathrm{cm}}^{3}$ and $T_{mbulk}=600.61\;K$ [50]) as seen in Figure 3. The first experimental set of melting points of Au nanoparticles are carried out for Au nanoparticles deposited on an amorphous carbon substrate [2], where the second experimental set is for silica-encapsulated Au nanoparticles [5]. The first experimental sets of melting point of Pb are carried out for Pb nanoparticles that were deposited on carbon substrate [6], while they were deposited on silicon monoxide substrate in the second experimental set [7]. The experimental values of the melting point of spherical Au and Pb nanoparticles (in an FCC structure) are given by their diameters; therefore, the relation $n=0.74{\left(D/d\right)}^{3}+1.82{\left(D/d\right)}^{2}$ [27,28] is used to calculate the corresponding number of atoms n. The calculated melting points based in the present model agree with the experimental sets of Au and Pb nanoparticles containing n≥1000 atoms. The deviations between the calculated melting points for spherical nanoparticles and the first experimental sets of Au nanoparticles containing n<1000 and Pb nanoparticles containing n<6000 atoms are due to the effect of their substrates [28,51]. The agreement of the calculated melting points of spherical nanoparticles with experimental values shows that the present model is effective in predicting the melting points.

The deviation between the experimental data and the calculated melting points based on the L-J potential in Qi et al.’s model [34] is because the shape effect is not considered. However, if the shape effect is considered, as in the present model, then the calculated melting points based on the L-J potential agree with the experimental data. Therefore, the deviation problem in Qi et al.’s model [34] is not due to the L-J potential. The surface-to-volume atomic ratio parameter $s_{n}$ that depends on the shape of the nanoparticles plays an important role in the present model. The parameter $s_{n}$ allows the surface atoms to have a larger effect than the interior atoms in the nanoparticles when their sizes reduce. Therefore, the calculated melting points of the nanoparticles based on the present model are smaller than that predicted by Qi et al.’s model [34] and agree with experimental data. The deviation between the calculated melting points based on the present model and the experimental values of melting points, as discussed above, when n<1000 for Au nanoparticles and n<6000 for Pb nanoparticles, is due the environment’s effect on the nanoparticles. However, the agreement between the calculated and experimental values is perfect if the environmental effect is reduced, as in the silica-encapsulated Au nanoparticles when n<1000 because they are considered as individual nanoparticles [5], as seen in Figure 3.

**Figure 4:**
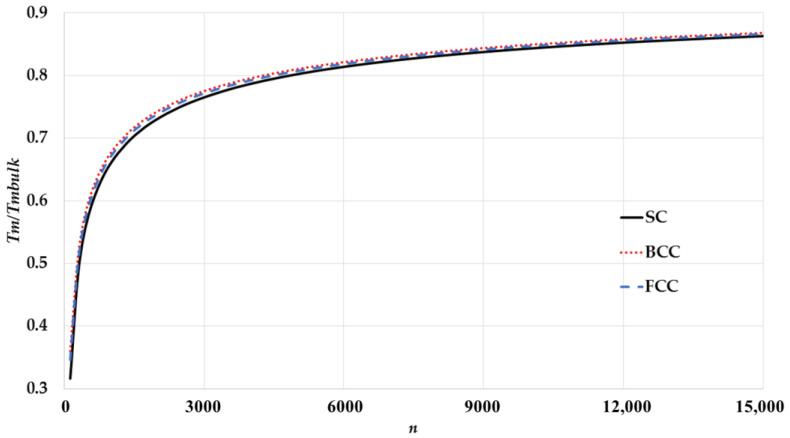
The variation of ratio $T_{m}/T_{mbulk}$ as function of the number of atoms n of spherical nanoparticles in different cubic structures.

The calculated melting points of spherical nanoparticles for different cubic structures are compared in Figure 4. The calculations show that the structure effect on the melting points is obvious for small nanoparticles because of the appearance of the lattice structure parameter $a_{ij}$ and surface-to-volume atomic ratio parameter $s_{n}$ in the potential parameters.

The Qi and Wang model showed that the melting point $T_{m}$ of a nanoparticle is linearly proportional to the reciprocal of the nanoparticle size D [24,25]:

(18) $$\frac{T_{m}}{T_{mbulk}}=T_{mbulk}\left(1-\frac{3P_{f}d}{P_{L}D}\right)$$

The constant C in Equation (1) equals to $3P_{f}d/P_{L}$. However, using Equation (4), then Equation (18) to get:

(19) $$\frac{T_{m}}{T_{mbulk}}=1-\frac{3P_{f}}{P_{L}^{\frac{2}{3}}n^{\frac{1}{3}}}$$

**Figure 5:**
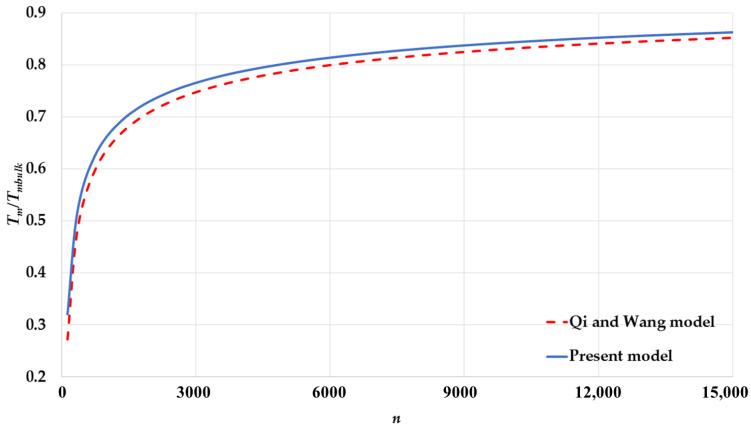
The variation of ratio $T_{m}/T_{mbulk}$ as function of number of atoms n of spherical nanoparticles in an SC structure using the present model and compared to Qi and Wang model [24].

**Figure 6:**
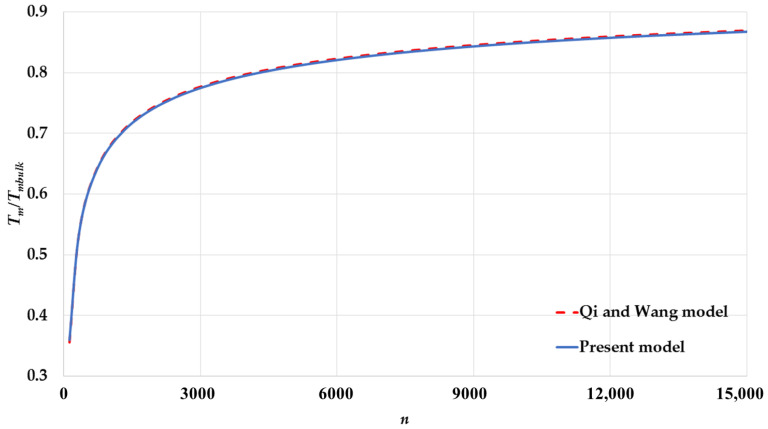
The variation of ratio $T_{m}/T_{mbulk}$ as function of number of atoms n of spherical nanoparticles in a BCC structure using the present model and compared to Qi and Wang model [24].

**Figure 7:**
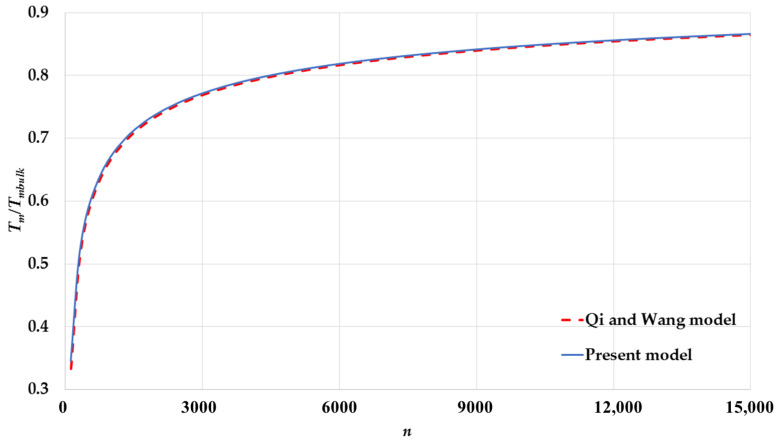
The variation of ratio $T_{m}/T_{mbulk}$ as function of number of atoms n of spherical nanoparticles in an FCC structure using the present model and compared to Qi and Wang model [24].

The calculated melting points by Equation (17) based on the L-J potential function are compared to the calculated melting points by Equation (19) based on Qi and Wang’s model [24,25] for spherical nanoparticles having different cubic structures: Figure 5 for an SC, Figure 6 for a BCC, Figure 7 for an FCC. The calculated melting points by both models show an excellent agreement for spherical nanoparticles with BCC and FCC structures and a small deviation for an SC structure. The present model based on the L-J potential function predicts that the melting point $T_{m}$ of nanoparticles is linearly proportional to the reciprocal of the nanoparticle size D. The present model provides a microscopic point view on the relation of the melting points of nanoparticles with their sizes, as discussed above, and shows how the contributions of the surface atoms and interior atoms play an important role in the instability of the nanoparticles core when their sizes are reduced.

**Figure 8:**
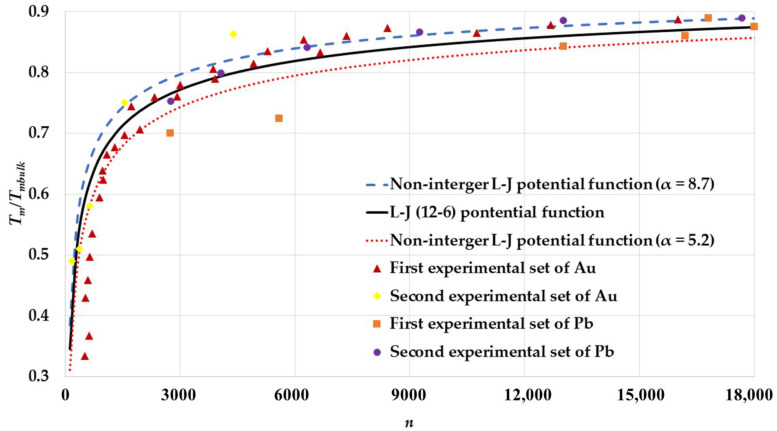
The variation of ratio $T_{m}/T_{mbulk}$ as function of number of atoms n of spherical nanoparticles in an FCC structure based on the non-integer L-J potential functions for different values of α parameter and compared to two different experimental sets of Au nanoparticles: The first experimental set of Au [2] and the second experimental set of Au [5], and two different experimental sets of Pb nanoparticles: The first experimental set of Pb [6] and the second experimental set of Pb [7].

A non-integer L-J potential function was proposed to calculate the cohesive energy and the melting point of nanoparticles [32], which takes the following form:

(20) $$U_{ij}=D_{0}\left\{{\left(\frac{r_{0}}{r_{ij}}\right)}^{2\alpha }-2{\left(\frac{r_{0}}{r_{ij}}\right)}^{\alpha }\right\}$$

where α is a dimensionless non-integer parameter. It was found that if the α parameter is small, then the nanoparticle becomes stable with small values of cohesive energy [32]. The non-integer L-J potential function with different values of α=5.2, 6 (L-J (12-6) potential function), and 8.7 is used to calculate the melting points of spherical nanoparticles in an FCC structure and compare to the two experimental sets of Au nanoparticles melting points [2,5] and the two experimental sets of Pb nanoparticles melting points [6,7] as seen in Figure 8. As shown in the figure, most of the experimental data are lying between the calculated melting point curves when α=5.2 and 8.7. This result may explain the deviation of some experimental data from the calculated melting point based on the L-J potential due to the environment’s effect. The environment’s effect may have an effect on the form of repulsive and attractive interactions in potential function. Therefore, the repulsive potential $\propto r^{-12}$ and the dipole attractive potential $\propto r^{-6}$ in the L-J potential can be replaced by an effective repulsive potential $\propto r^{-2\alpha }$ and an effective attractive potential $\propto r^{-\alpha }$ to introduce the effect of the nanoparticle’s environment.

## 4. Conclusions

A model is built to calculate the melting point of nanoparticles based on the sum of all energy bonds between the atoms in an equilibrium configuration based on L-J (12−6) and the non-integer L-J potential functions. The model considers the size, the shape, and the structure of the nanoparticles, and considers the dangling bonds of the surface atoms of the nanoparticles. The calculated melting points for spherical nanoparticles agree with the experimental data of Au and Pb nanoparticles and Qi and Wang’s model [24,25]. The agreement of the present model with the experimental values is due to the existence of the surface-to-volume atomic ratio parameter $s_{n}$ that depends on the shape of the nanoparticles and plays an important role in the contribution of the surface atoms in the nanoparticles when their sizes are reduced. The deviation between the experimental data and the calculated melting points based on the L-J potential is not due to the L-J potential; instead, it is due to whether the model considers the shape effect or not, as in Qi et al.’s model [34].

The calculated melting points based on the L-J (12-6) potential function for spherical nanoparticle follow the Gibbs-Thompson relation. However, the present model provides a microscopic description of the variation of the thermal properties of nanoparticles with their sizes. Using the non-integer L-J potential function can explain the deviation of the experimental data for Au and Pb nanoparticles from the calculated melting points based on L-J potential. The terms of the potential function can be considered as an effective repulsive potential $\propto r^{-2\alpha }$ and an effective attractive potential $\propto r^{-\alpha }$ instead of Pauli’s repulsive potential $\propto r^{-12}$ and dipole attractive potential $\propto r^{-6}$ to introduce the environmental effect.

## Figures and Tables

**Table 1:** The values of the volume and the surface packing factors for different cubic structures [27].

Cubic Structure	$P_{L}$	$P_{f}$
FCC	$\frac{\pi }{3\sqrt{2}}$	$\frac{\pi }{\sqrt{3}}$
BCC	$\frac{\pi }{6\sqrt{2}}$	$\frac{3\pi }{8\sqrt{2}}$
SC	$\frac{\pi }{6}$	$\frac{\pi }{4}$

**Table 2:** The values of $A_{6}^{’}$ and $A_{12}^{’}$ for different cubic structures [38].

Cubic Structure	$A_{6}^{’}$	$A_{12}^{’}$
FCC	14.45	12.13
BCC	12.25	9.11
SC	8.40	6.20

## Data Availability

The study did not report any data.
